# In-Silico Patterning of Vascular Mesenchymal Cells in Three Dimensions

**DOI:** 10.1371/journal.pone.0020182

**Published:** 2011-05-25

**Authors:** Tal Danino, Dmitri Volfson, Sangeeta N. Bhatia, Lev Tsimring, Jeff Hasty

**Affiliations:** 1 Department of Bioengineering, University of California San Diego, La Jolla, California, United States of America; 2 Biocircuits Institute, University of California San Diego, La Jolla, California, United States of America; 3 Molecular Biology Section, Division of Biological Science, University of California San Diego, La Jolla, California, United States of America; 4 Department of Electrical Engineering and Computer Science, Massachusetts Institute of Technology, Cambridge, Massachusetts, United States of America; Tata Institute of Fundamental Research, India

## Abstract

Cells organize in complex three-dimensional patterns by interacting with proteins along with the surrounding extracellular matrix. This organization provides the mechanical and chemical cues that ultimately influence a cell's differentiation and function. Here, we computationally investigate the pattern formation process of vascular mesenchymal cells arising from their interaction with Bone Morphogenic Protein-2 (BMP-2) and its inhibitor, Matrix Gla Protein (MGP). Using a first-principles approach, we derive a reaction-diffusion model based on the biochemical interactions of BMP-2, MGP and cells. Simulations of the model exhibit a wide variety of three-dimensional patterns not observed in a two-dimensional analysis. We demonstrate the emergence of three types of patterns: spheres, tubes, and sheets, and show that the patterns can be tuned by modifying parameters in the model such as the degradation rates of proteins and chemotactic coefficient of cells. Our model may be useful for improved engineering of three-dimensional tissue structures as well as for understanding three dimensional microenvironments in developmental processes.

## Introduction

The evolution of tissue form in development, wound healing, and regeneration is a dynamic process that involves the integration of local cues on cell fate and function. These cues include interactions with soluble factors (growth factors, morphogens, dissolved gases) and insoluble factors (extracellular matrix, neighboring cells) in a three-dimensional context. A fundamental understanding of how tissue structure evolves is critical to the rational development of engineered tissues for therapeutic applications. There has been increasing evidence that culture of cells in three-dimensions compared to two-dimensions can dramatically impact cellular organization, polarity, and drug responsiveness[1]–[7]. Here we sought to isolate the role of diffusion/reaction gradients in three dimensions while excluding morphogenetic effects.

Although there have been several modeling efforts to study cell pattern formation and organization in two dimensions[8]–[18], there has not been much attention devoted to three-dimensional systems[3], [19]. Recently, a phenomenological two dimensional reaction-diffusion model with morphogen identified as Bone Morphogenic Protein 2 (BMP-2) and inhibitor Matrix Gla Protein (MGP) was shown to produce the patterning of human vascular mesenchymal cells[11]. Using a first-principles approach we derive a model based on the underlying biochemical interactions of BMP-2 and MGP and show that our model produces similar patterns as two dimensional experiments. We then perform simulations with our model in three dimensions and explored the types of patterns observed and effect of model parameters. We find that the patterns seen in three dimensions are strikingly different than those seen in two-dimensions and we examine their stability numerically. We discuss these findings in the context of engineering desired tissue structures and also relate to the important differences seen in cell organization between two and three dimensional settings.

**Figure 1:**
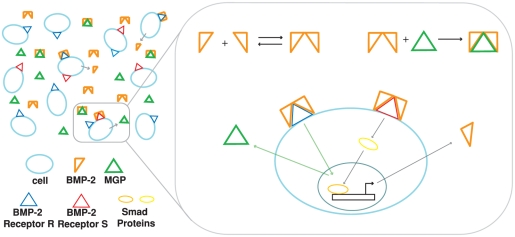
Diagram showing interactions between BMP-2, MGP, and cells in culture. The binding of a BMP-2 dimer to receptors R and S stimulates production of BMP-2 and MGP, while the inding of MGP to BMP-2 outside of the cell prevents this process. The production of BMP-2 occurs via the Smad signalling pathway and the production of MGP occurs through an unknown pathway.

The morphogen in the model is Bone Morphogenic Protein 2 (BMP-2), a member of the TGF-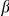 superfamily which to date has over 20 members[20], [21]. BMP-2 is able to dimerize to its biologically active form [26 kDa for the dimer] and is a potent stimulator of cells to differentiate to an osteoblast-like fate. This occurs through the binding of a BMP-2 dimer to a TGF-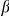 receptor complex, which then functions to phosphorylate the Smad proteins. These proteins then translocate to the nucleus and act as transcription factors for various genes including the gene for BMP-2[11], [22]. In addition, BMP-2 has been shown to be a strong chemoattractant for these cells and thus is a good candidate for a morphogen in the reaction-diffusion model [11], [23]. MGP is a smaller (10.4 kDa) regulatory protein for BMP-2. MGP is thought to inactivate BMP-2 by physical binding to BMP-2 and prevent binding to the receptors [24]–[31]. The presence of BMP-2 also stimulates production of MGP through an unknown mechanism[11], [32]. In Fig. 1, an illustration of the system is shown with the relevant biochemical reactions.

Our simplified model for the reaction-diffusion process of the vascular mesenchymal cell system is derived from the underlying biochemical reactions. The reactions for BMP-2, MGP, and BMP-2 Receptor complexes on the surface of cells are shown schematically in Fig. 1. Transcription, translation, and export out of the cell for BMP-2 and MGP were lumped together for simplicity. We simplified the model using a multiple time scale analysis, which takes advantage of the difference in time scales between the kinetic processes and assumes a local quasi-equilibrium. Below, the model equations are presented in a scaled form with dimensionless concentrations of BMP-2 (U), MGP (V), and cells (n) as functions of space (x,y,z) and time (t). The derivation of the model can be found in the Supplementary Info.

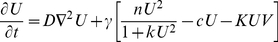
(1)

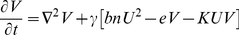
(2)

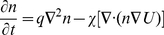
(3)


In the first equation, the first term on the r.h.s represent diffusion of BMP-2, the second term represents an autocatalytic production of BMP-2 that saturates, the third term is a degradation of BMP-2 at rate *c*, and the fourth is a nonlinear degradation by physical binding of BMP-2 to MGP. The equation for MGP has a similar diffusion term as well as production by BMP-2 term which is known not to saturate[11], [27], degradation of MGP at rate *e*, and nonlinear degradation by physical binding of BMP-2 to MGP. The equation for cell concentration (n) has a diffusion term as well as chemotaxis term that accounts for cells movement toward higher regions of chemoattractant (BMP-2) and also depends on cell density. Parameters D = 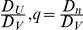 are the ratios of diffusion coefficients for BMP-2 to MGP, Cells to MGP, respectively. The coefficient *b* represents the relative production of MGP to BMP-2, *c* and *e* represent the degradation of U and V, and *K* represents the nonlinear degradation of U and V by physical binding. The parameter 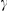 is a scaling parameter for the relation between domain size and chemical kinetics.

The diffusion coefficients, production rate of BMP-2, degradation rates of BMP-2 and MGP were taken from the literature [11], [33]. The production of MGP is known to be similar to BMP-2 (although its value uncertain) and was set to a value of 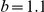. The nonlinear degradation coefficient, 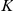, can be expressed in terms of kinetic rate parameters but these rates are also unknown, and thus was set to 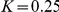 along with 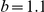 to reproduce the stripe patterns seen in previous work[11]. The mean cell density n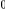, which is conserved in the dynamics is set to n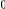 = 1.

## Results

**Figure 2:**
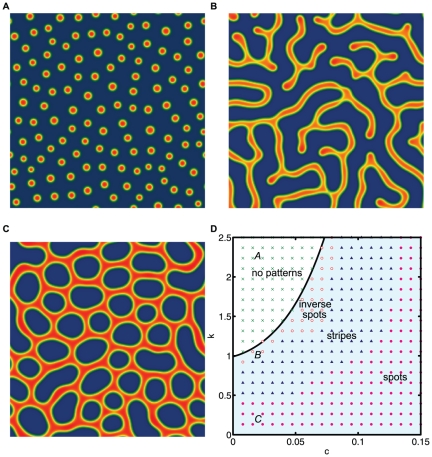
2D steady state patterns of cells. The derived model shows (a)spots(k = 0.2,c = 0.12), (b)stripes(k = 0.7,c = 0.04), and (c) inverse spots(k = 0.95,c = 0.005) by varying k and c. The parameters used were D = 0.005, q = 0.003, K = 0.25, B = 1.1, *γ*  = 600 and the box length of the simulation is equivalent to 1 cm. Red color indicates higher cell density while blue indicates low.

**Figure 3:**
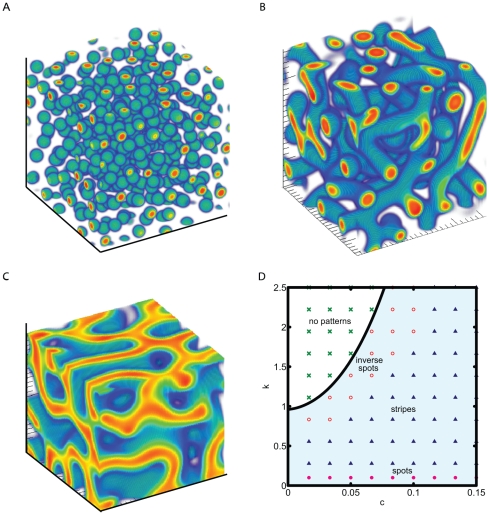
3D steady state patterns of cells. The derived model shows spherical spots(k = 0.2,c = 0.12), tubes(k = 0.2,c = 0.04), and sheet-like structures(k = 0.8,c = 0.04) by varying k and c. The parameters used were D = 0.005, q = 0.003, K = 0.25, B = 1.1, *γ*  = 600 and the box length of the simulation is equivalent to 1 cm. The lowest values were made transparent for clarity while red color indicates higher cell densitywhile blue indicates low.

The mathematical model admits up to 3 real uniform steady states for the parameter region we explored. Of these, one is always the zero solution{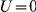,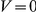,n = 1}, the other is low {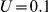,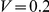,n = 1}, and the third is high {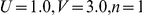}. In the supplementary info, a linear stability analysis was carried out to analyze the stability of these steady states and determine the region where patterns are found. Briefly, the linear stability analysis analyzes a small perturbation from the steady state and determines which modes of the perturbation are unstable, which generally corresponds to the size of the perturbation. Among these states, the zero solution is always stable and the low solution is always unstable. The high state is stable with respect to spatially uniform perturbations, but it can be unstable with respect to spatially non-uniform modes. We performed simulations and analyzed the stability of these steady states (Supplementary info) and found that only the higher steady state produced patterns that resembled the experiments and is likely the physiologically relevant one. We start with an initial condition at this steady state and add a 1% relative random noise to model cell variation[11]. The simulations shown in Figures 2 and 3 are the state distribution of cells with red color indicating high levels of cell density and blue levels indicating low levels of cell density. The lowest values of cell density are made transparent for visual clarity. The parameters used unless otherwise specified were D = 0.005, q = 0.003, 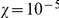, K = 0.25, B = 1.1, 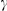 = 600 and the box length of the simulation is equivalent to 1 cm.

Simulations in two dimensions varying the parameters 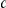 (degradation of BMP-2) and 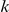 (saturation of production of BMP-2) are shown in Figure 2. Three basic types of steady state patterns emerge from the model (Fig. 2a–c): (a) spots, (b) stripes, and (c) inverse spots. By stripe patterns we mean that cells arrange in higher densities along stripe regions with characteristic thickness. The spot patterns correspond to clusters of cells and the inverse spots show connected structures of cells with gaps of no cells in between. The stripe and spot patterns were previously seen in the experimental two-dimensional setting, although the inverse spot patterns were not. Fig. 2(d) shows where the patterns are found in parameter space upon scanning parameters 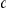 and 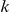. The solid line between the regions of no patterns and patterns is predicted by our linear stability analysis and matches with our visual inspection of the simulations. We used a 20×20 grid of numerical simulations and visually inspected the simulations to determine their pattern type. In regions that show existence of more than one pattern we labeled the pattern type by the majority of the pattern seen.

In Fig. 3, we show the simulations in three dimensions varying the same parameters 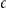 and 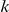. In three dimensions, the steady state patterns produced are (a) spheres of cells, (b) solid tubes, and (c) highly interconnected tubes which have planar surfaces. These three pattern types are somewhat analogous to the 2D patterns of spots, stripes and inverse spots, respectively. Movies for each of these cases can be found in the supplementary info(Supplementary Movies S1, S2, S3). The distinguishing feature between types (b) and (c) is that the cross section of the sheet like structures resemble stripes while the cross section of the solid tubes resembles spots. Fig. 3(d) also shows where the patterns are found in parameter space with a 9×9 grid of numerical simulations.

**Figure 4:**
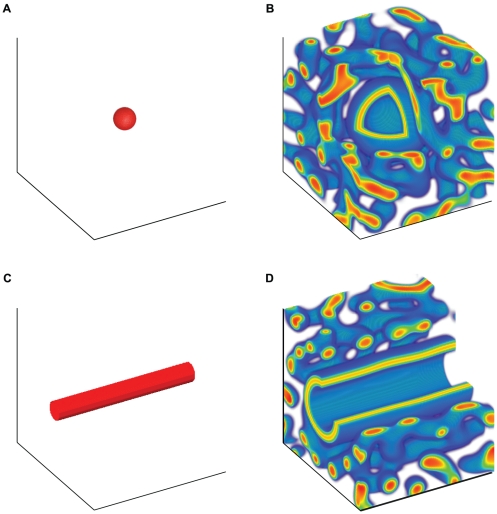
Initial and steady state patterns of cells produced by exogenous BMP-2. An initial condition of 2× higher concentration of BMP-2 is placed along the center (a) sphere or (b) cylinder and the cells are allowed to reach steady state. The stripe regime parameters were used and set as D = 0.005, q = 0.003, K = 0.25, B = 1.1, k = 0.7, c = 0.14, *γ*  = 600 with simulation box length set to 2 cm. The lowest values were made transparent for clarity while red color indicates higher cell density while blue indicates low. A cut of the simulation box in (a) 1/8 of cube and (b) 1/4 of cube was sliced out for easier visualization.

Fig. 4 shows the evolution of cells with an initial condition of a (a) spherical or (b) cylindrical region along the center axis containing at 2× higher BMP-2 concentration than the steady state. The surrounding region was set to the zero value. The parameters set for these simulations were those in the stripe pattern regime to mimic the previous experimental setting[11].

## Discussion

Figures 2(d) and 3(d) show the locations of the types of patterns in two dimensions and three dimensions as a function of parameters 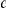 and 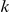. We see that in the two-dimensional case the spot patterns are seen over a wide range of parameters while in three-dimensional case these patterns are only rarely seen. In trying to correlate the 2D pattern region with the 3D pattern region we scaled the diffusion and chemotactic coefficient by 3/2 to reflect the change from 2D to 3D. We found that this did not significantly alter where the patterns are seen in the parameter space. This difference in the pattern location may arise because of the spatial symmetry of the problem. For instance, the tubes which are seen often in three-dimensions can be cut along different axes to form either the spot or stripe patterns seen in two-dimensions. Thus, they occupy a larger region in the parameter space for three-dimensions than in two-dimensions. For an experimental system with fixed parameters, we would predict that the organization of cells in two dimensions greatly differs from that in three dimensions, suggesting a possible reason for the biological differences seen in experimental culture of mammalian cells[1].

In the parameter space we explored, we found that multiple patterns can coexist for a fixed set of parameters and we examined the stability of each type. We ran a 2D simulation to steady state which showed only spots (point C, Figure 2d), and then increased the parameter *k* slowly while allowing the system to equilibrate. Doing this from point C to point B in Figure 2d we found that the spot patterns remained stable throughout the region and finally disappeared when reaching the no pattern region(point A). In the regions where stripes were found(point B), the spot patterns would temporarily nucleate into stripes and then go back to their spot pattern state. We also performed the opposite case starting at point B and decreasing *k*. In this case we found the patterns to go from the inverse spot pattern type to the stripe pattern, but then we found that at point C the cells remained in the stripe pattern type and did not change into the spot pattern type. This indicates that the inverse spot type of pattern is least stable to perturbations, while the stripe and spot patterns are more stable. Along with the fact that the inverse spot type is seen least in parameter space, this may suggest why this type of pattern has been difficult to realize experimentally[11].

We also performed simulations that can be directly tested in three-dimensional experiments. For instance, an experiment where a higher concentration of BMP-2 is produced at the center region can be represented by an analogous initial condition in our simulation. In Fig. 4, simulations were performed with an initial condition set so that a local (a) sphere or (b) cylindrical region of BMP-2 is at a 2× higher concentration than the steady state value(see Supplementary Movies S1, S2, S3). The parameters set for these simulations were those in the stripe pattern regime to mimic previous experimental observations for the vascular mesenchymal cell system. For the spherical case, we found that the morphogen concentration will grow in expanding spheres and the cells will arrange themselves in the same way. For the cylindrical initial condition, we found that the cells will evolve in a hollow cylinder from the initial condition forming a vessel-like shape.

Additionally, we investigated the effect of cell parameters on the patterns observed. The random cell motility, 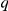, and the chemotactic coefficient, 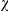, both play a role in the stability and pattern selection of cells. We found that by varying the ratio of 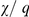, it is possible to change the pattern type from one to another and it is possible to end up in a regime where no patterns are formed. This situation occurs for points near the stability border with a change to the nominal value of 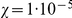. When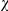 is changed to 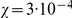 and then 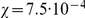 the patterns observed are of the inverse spot and stripe pattern type, respectively(Supplementary info). For the higher ratio of 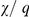, we found that the cells are more often found in the spot pattern type, showing that these are most stable types(Supplementary Info).

The simulations we have done here show the importance of three-dimensional modeling of cell organization. In three dimensions we found that the patterns and organization of cells is much richer than in 2D and found that the same model system with fixed parameters in two and three-dimensions can exhibit different steady-state pattern types. Simulations to mimic developmental processes and engineering of three-dimensional tissue structures will thus find these techniques to be useful for predicting cell organization in three dimensions. In addition, we presented simulations that could easily be tested in two- or three- dimensional experiments to validate our model.

## Materials and Methods

We performed two- and three- dimensional simulations using a pseudospectral technique as described in[34]. The method handles the nonlinearities explicitly in real space and diffusion in Fourier space. To simulate the cell equation we kept the zero mode a constant since the total cell mass is conserved. We found that the method shows agreement up to numerical accuracy with solutions to known nonlinear equations (Supplementary info). Furthermore, we saw convergence of our numerical results for a range of timesteps and spatial discretizations. The technique we used assumes periodic boundaries on the spatial domain.

Three-dimensional simulations were parallelized using the Message Passing Interface (MPI 2.0) in conjunction with the FFTW library. We used a 256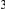 (a 128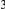 for the 9×9 scan in Figure 3) with 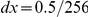 which typically required about 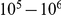 steps to reach steady state at a step size of dt = 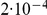. For the 256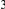 grid, a typical computation time of 120 hours on a single processor or 30 hours on eight processors was needed to perform most simulations. IDL software (ITT Visual Information Solutions) was used for visualizing three-dimensional graphics.

## Supporting Information

Movie S1 Simulation showing formation of spot patterns in three-dimensions.
(AVI)

Movie S2 Simulation showing formation of inverse spot patterns in three-dimensions.
(AVI)

Movie S3 Simulation showing formation of tube patterns in three-dimensions.
(AVI)
